# Prevalence of *pvmrp1* Polymorphisms and Its Contribution to Antimalarial Response

**DOI:** 10.3390/microorganisms10081482

**Published:** 2022-07-22

**Authors:** Yi Yin, Gangcheng Chen, Myat Htut Nyunt, Meihua Zhang, Yaobao Liu, Guoding Zhu, Xinlong He, Fang Tian, Jun Cao, Eun-taek Han, Feng Lu

**Affiliations:** 1 Department of Pathogenic Biology, School of Medicine, Yangzhou University, Yangzhou 225009, China; yinyi523@163.com (Y.Y.); hexl@yzu.edu.cn (X.H.); ftian@yzu.edu.cn (F.T.); 2 Key Laboratory of National Health Commission on Parasitic Disease Control and Prevention, Jiangsu Provincial Key Laboratory on Parasite and Vector Control Technology, Jiangsu Institute of Parasitic Diseases, Wuxi 214064, China; eddiecgc197@163.com (G.C.); zmh-07@163.com (M.Z.); liuyaobao@jipd.com (Y.L.); jipdzhu@hotmail.com (G.Z.); caojun@jipd.com (J.C.); 3 Department of Medical Research, Ministry of Health and Sports, Yangon 11191, Myanmar; drmhnyunt@gmail.com; 4 Department of Medical Environmental Biology and Tropical Medicine, School of Medicine, Kangwon National University, Chuncheon 24341, Gangwon-do, Korea; 5 Affiliated Hospital of Yangzhou University, Yangzhou 225000, China; 6 Jiangsu Key Laboratory of Experimental & Translational Non-Coding RNA Research, School of Medicine, Yangzhou University, Yangzhou 225009, China; 7 Jiangsu Key Laboratory of Zoonosis, Jiangsu Co-Innovation Center for Prevention and Control of Important Animal Infectious Diseases and Zoonoses, Yangzhou 225009, China

**Keywords:** *Plasmodium vivax*, *pvmrp1*, genetic diversity, natural selection

## Abstract

As more sporadic cases of chloroquine resistance occur (CQR) in *Plasmodium vivax* (*P. vivax*) malaria, molecular markers have become an important tool to monitor the introduction and spread of drug resistance. *P. vivax* multidrug resistance-associated protein 1 (PvMRP1), as one of the members of the ATP-binding cassette (ABC) transporters, may modulate this phenotype. In this study, we investigated the gene mutations and copy number variations (CNVs) in the *pvmrp1* in 102 *P. vivax* isolates from China, the Republic of Korea (ROK), Myanmar, Papua New Guinea (PNG), Pakistan, the Democratic People’s Republic of Korea (PRK), and Cambodia. And we also obtained 72 available global *pvmrp1* sequences deposited in the PlasmoDB database to investigate the genetic diversity, haplotype diversity, natural selection, and population structure of *pvmrp1*. In total, 29 single nucleotide polymorphisms reflected in 23 non-synonymous, five synonymous mutations and one gene deletion were identified, and CNVs were found in 2.9% of the isolates. Combined with the antimalarial drug susceptibility observed in the previous in vitro assays, except the prevalence of S354N between the two CQ sensitivity categories revealed a significant difference, no genetic mutations or CNVs associated with drug sensitivity were found. The genetic polymorphism analysis of 166 isolates worldwide found that the overall nucleotide diversity (π) of *pvmrp1* was 0.0011, with 46 haplotypes identified (*Hd* = 0.9290). The ratio of non-synonymous to synonymous mutations (dn/ds = 0.5536) and the neutrality tests statistic Fu and Li’s D* test (Fu and Li’s D* = −3.9871, *p* < 0.02) suggests that *pvmrp1* had evolved under a purifying selection. Due to geographical differences, genetic differentiation levels of *pvmrp1* in different regions were different to some extent. Overall, this study provides a new idea for finding CQR molecular monitoring of *P. vivax* and provides more sequences of *pvmrp1* in Asia for subsequent research. However, further validation is still needed through laboratory and epidemiological field studies of *P. vivax* samples from more regions.

## 1. Introduction

*Plasmodium vivax* was responsible for up to 4.5 million cases of malaria in 2020 [1]. Although it is rarely fatal, *P. vivax* malaria is the leading cause of malaria-related deaths outside of Africa [2]. In most vivax-endemic areas, a combination of chloroquine (CQ) is the first-line treatment for uncomplicated vivax malaria. Despite inexpensive, well tolerated, and widely available chloroquine resistance (CQR), vivax malaria was first reported from Papua New Guinea (PNG) in 1989 [3], and has been documented in more than ten countries [4], especially in multiple regions of Myanmar [5,6,7]. *P. vivax* resistance to other antimalarial drugs, such as mefloquine (MQ), sulfadoxine-pyrimethamine (SP) and primaquine (PQ), has also been reported widely [8,9]. The rise of these drug-resistant parasites threatens the global efforts to control malaria. However, molecular markers of drug resistance in *P. vivax* remain elusive [10]. 

Generally, genetic variation in the expression of transporter proteins could contribute to evading antimalarial action [11]. ATP-binding cassette (ABC) transporters are transmembrane proteins that can carry various substrate types, such as drugs and metabolic products [12]. The overexpression or mutation of many ABC transporters can lead to drug resistance [13]. As one of the members of the ABC subfamily C, the multidrug resistance-associated proteins (MRPs) are associated with antimalarial resistance. The increased expression of *pfmrp1* has been associated with the resistance of MQ and CQ, and gene polymorphisms in *pfmrp1* with in vivo selection after SP and artemether/lumefantrine treatment [14,15,16]. Furthermore, the deletion of this gene in the CQR *P. falciparum* strain results in increased sensitivity to CQ, Quinine (QN), artemisinin, piperaquine (PPQ) and PQ [17]. Since *P. vivax* cannot be cultured continuously in vitro [18], most research on the molecular mechanism of drug resistance in *P. vivax* has focused on the homologous genes related to the drug resistance of *P. falciparum* [10,19]. Hence, we chose to study the association between *pvmrp1* and the development of *P. vivax* resistance to antimalarial drugs.

In this study, we genotyped 94 *P. vivax* isolates from China, the Republic of Korea (ROK), Myanmar, PNG, Pakistan, the Democratic People’s Republic of Korea (PRK), and Cambodia, and combined the 72 available global *pvmrp1* sequences deposited in the PlasmoDB database to analyze the characterization of genomic variation and population genomics methods, including genetic differentiation, haplotype network, linkage disequilibrium (LD) and the phylogenetic tree. Meanwhile, the correlation between *pvmrp1* and antimalarial drug susceptibility observed in the previous in vitro assays was further analyzed.

## 2. Materials and Methods

### 2.1. Study Sites and Participants

Clinical blood samples with *P. vivax* infections (*n* = 102) were obtained from seven countries, including China (*n* = 46), ROK (*n* = 27), Myanmar (*n* = 21), PNG (*n* = 3), Pakistan (*n* = 3), PRK (*n* = 1), and Cambodia (*n* = 1). The Chinese samples were collected from local hospitals or centers for disease control and prevention in central China from 2005 to 2008 [20]; The South Korean samples were from local hospitals in endemic areas, such as the ROK, from 2007 to 2009 [21]; The samples of Myanmar were collected from Wet-Won Station Hospital, Yangon, Myanmar, in 1999 [21]; Other samples of *P. vivax* isolates were imported malaria cases in China. The protocol was reviewed and approved by the National Institute of Parasitic Diseases, the Chinese Center for Disease Control and Prevention, the Kangwon National University Hospital Human Ethics Committee, and the Myanmar Department of Health. The sequence of *pvmrp1* from 72 *P. vivax* isolates deposited in the PlasmoDB database were downloaded and analyzed, and originated from 10 countries, including South America: Columbia (*n* = 21), Peru (*n* = 16), Mexico (*n* = 13), and Brazil (*n* = 3); Southeast Asia: Myanmar (*n* = 4) and Thailand (*n* = 7); Oceania: PNG (*n* = 4); East Asia: PRK (*n* = 1); South Asia: India (*n* = 2) and Africa: Mauritania (*n* = 1). All sample information was listed in Spreadsheet S1, and all sequences have been uploaded to Genbank with accession numbers from ON933478 to ON933571.

### 2.2. Gene Sequence and Protein Structure Prediction

The gene sequence of *pvmrp1* (PVX_097025, *Sal*-I reference genome) was retrieved from PlasmoDB database (http://www.plasmodb.org, accessed on 11 December 2021). The conserved domains of PvMRP1 were searched within the NCBI’s Conserved Domain Database (https://www.ncbi.nlm.nih.gov/cdd/, accessed on 20 September 2021) [22]. Protein domains, transmembrane domains (TMDs), and nucleotide-binding domains (NBDs) were predicted by the Simple Modular Architecture Research Tool (http://smart.embl-heidelberg.de/, accessed on 20 September 2021) [23] and TMHMM server 2.0 (https://services.healthtech.dtu.dk/service.php?TMHMM-2.0, accessed on 20 September 2021), respectively [24]. 

### 2.3. Single Nucleotide Polymorphisms (SNPs) Identification in pvmrp1 Gene

According to the manufacturer’s instructions, genomic DNAs from the 102 whole blood samples were individually extracted using a QIAamp DNA blood kit (Qiagen, Valencia, CA, USA) and were stored at −20 °C in the previous studies. The *pvmrp1* gene was amplified by nested or semi-nested PCR using specific primers (Table 1). All reactions were performed in 20 µL containing 4 µL of 5× Phusion HF Buffer (7.5 mM Mg^2+^ plus), 0.2 mM of each dNTP, 0.25 µM of each outer primer, 0.4 U Phusion High Fidelity DNA Polymerase (New England Biolabs, Ipswich, MA, USA) and 1 µL of genomic DNA or the amplicon from the first PCR. The PCR was performed with initial denature at 98 °C for 30 s, followed by 35 cycles of 98 °C for 10 s, 56–61 °C for 30 s (Table 1), and 72 °C for 1.5 min, and a final extension period at 72 °C for 10 min. The second round of PCR amplification products was purified and sequenced by GenScript (Nanjing, China). The nucleotide sequences were compared and spliced using Lasergene software (DNASTAR, Madison, WI, USA). 

### 2.4. Determination of pvmrp1 Copy Number (CN)

The copy number variations (CNVs) of *pvmrp1* were measured by TaqMan-BHQ1 probe quantitative PCR assays performed on the Roche LC480 thermal cycler. A reference plasmid was constructed with *pvmrp1* (nt, 1762–1872) and *pvtubulin* (nt, 1644–1765) fragments in a ratio of 1:1 similar as described previously [21]. Probes and primers used for amplification of both genes were listed in Table 1. The probe target *pvmdr1* was labeled at the 5′ end with the FAM reporter dye, and *pvtubulin* was the HEX reporter dye, both of which were labeled at the 3′ end with the quencher dye BHQ1. A real-time PCR was conducted in 10 µL volumes containing 5 µL of Lightcycler^®^ 480 Probes Master 2 × (Roche Applied Science, Penzberg, Germany), 400 nM of each forward and reverse primer, 250 nM of each probe, and 1 µL of template DNA. Amplifications were performed in triplicate and the cycling parameters were as follows: 95 °C for 10 min, then 40 cycles of 95 °C for 15 s and 58 °C for 30 s. The single copy of the *pvtubulin* gene served as an internal control. The relative CN of *pvmrp1* was calculated using a relative standard curve method as normal, and the amplifications were repeated according to specifications [20,21]. 

### 2.5. Data Analysis

The multiple sequence alignments of worldwide isolates containing the wild reference sequence of *pvmrp1* were obtained using the MUSCLE in the MEGA v7.0.18 program to obtain SNPs [25,26]. Moreover, the average nucleotide diversity (π), haplotype diversity (*Hd*), and the neutrality tests (Tajima’s D test and Fu and Li’s D* test) were further analyzed to identify *pvmrp1* gene polymorphism and determined whether it is under the neutral evolution model [26,27], in order to evaluate the evolutionary relationship of the *pvmrp1* gene. The estimation of genetic differentiation (F_ST_) of the *pvmrp1* was analyzed. Haplotype network was also implemented to identify the genetic association of the *pvmrp1* haplotypes by means of the Median-Joining method in the NETWORK v5.0 program [28]. The phylogenetic tree of the aligned sequences was constructed using the Neighbor-Joining method in the MEGA v7.0.18 program [29]. In addition, pairwise LD of *pvmrp1* gene at different polymorphic sites was calculated using the DNASP v5.10.01 program [30]. Compared to the previous in vitro drug sensitivity test [20,21], Fisher’s exact test and chi-square analysis were performed to analyze whether it was related to the polymorphism of *pvmrp1*. As the sample size is too small to carry out statistical analysis, the data would not be displayed. A value of *p* < 0.05 was considered significant.

## 3. Results

### 3.1. Characterization of PvMRP1

**Figure 1:**
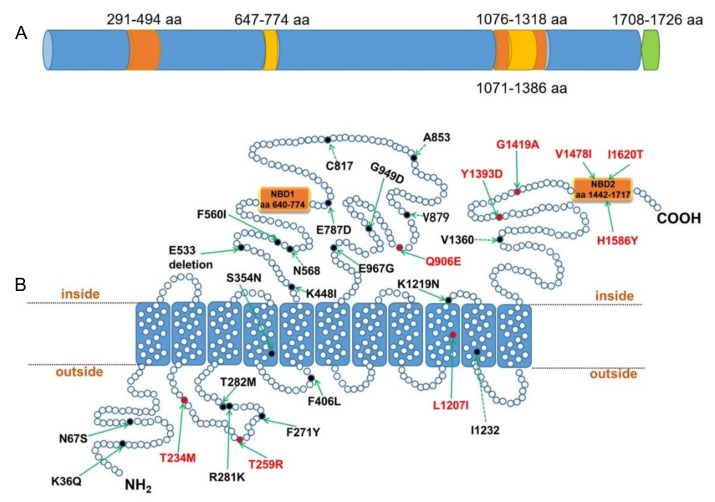
Predicted PvMRP1 primary protein structure and gene mutation. (**A**) Diagram of PvMRP1 primary protein structure, orange, yellow and green colors indicate conserved domain, Pfam domains, and GPI-anchor, respectively. (**B**) PvMRP1 shows the 11 predicted transmembrane helices. The positions of all the different mutations identified from the analysis of 94 geographically diverse isolates are indicated by filled circles, solid lines represent non-synonymous mutations, dotted lines represent synonymous mutations. The red font indicates that the mutation was reported previously [10].

As a member of the ABC superfamily, PvMRP1 was encoded by the intron-less gene *pvmrp1* (5181 bp), which represented a larger protein (ca. 1726 a.a., *Sal*-I reference genome). PvMRP1 has 2 TMDs including 11 transmembrane helices (TM) (aa 120–143, 168–191, 308–331, 343–366, 418–441,1065–1088, 1123–1146, 1160–1183, 1195–218, 1221–1237, and 1300–1323), and two NBDs (aa 640–774 and 1442–1717) (Figure 1B). In addition, some specific regions were also identified in the PvMRP1 predicted primary protein structure, such as two Pfam region (aa 646–774, 1076–1318), two conserved domains (aa 291–494 and 1071–1386) and a GPI-anchor (aa 1708–1726) (Figure 1A).

### 3.2. Identification of Gene Mutations and CN in pvmrp1 among Collected Blood Samples in Asia

Of the 102 *P. vivax* isolates, 94 samples (92.1%) were sequenced successfully for the *pvmrp1* gene, including China (*n* = 43), ROK (*n* = 27), Myanmar (*n* = 18), PNG (*n* = 2), Pakistan (*n* = 2), PRK (*n* = 1) and Cambodia (*n* = 1). Compared with *Sal*-I as the wild reference type, 29 polymorphic sites were observed, 23 (79.3%) of which resulted in non-synonymous mutations, one site E533 (GAA) deletion, and five (17.2%) synonymous mutations were identified. Compared with the previously reported SNPs [10], nine non-synonymous mutations were repeatable, and the present analysis revealed 20 different SNPs (Figure 1B). Three non-synonymous substitutions, including T259R (97.87%), Y1393D (97.87%) and V1478I (95.74%), were high prevalence and approached fixation. The wild type T259 was found in two isolates from Oceania, and the other two wild types were found in isolates from South Asia and East Asia (Table 2). Of the 29 mutations found in this study, 20 were shown to be region-specific, such as mutations R281K, S354N, E787D, A853, G949D, and V1360 which were only observed in East Asia (range: 12.7–38.0%), V879 and L1207I were unique to the Southeast Asia at high frequency (89.5%). Three mutations (T234M, Q906E and I1232) were more frequent in isolates from Southeast Asia compared to the other areas. Although only 2 isolates were from South Asia, four mutations (F271Y, T282M, F560I and G1419A) were identified exclusively. Also, two isolates were confirmed, I1620T specifically in Oceania (Table 2).

**Figure 2:**
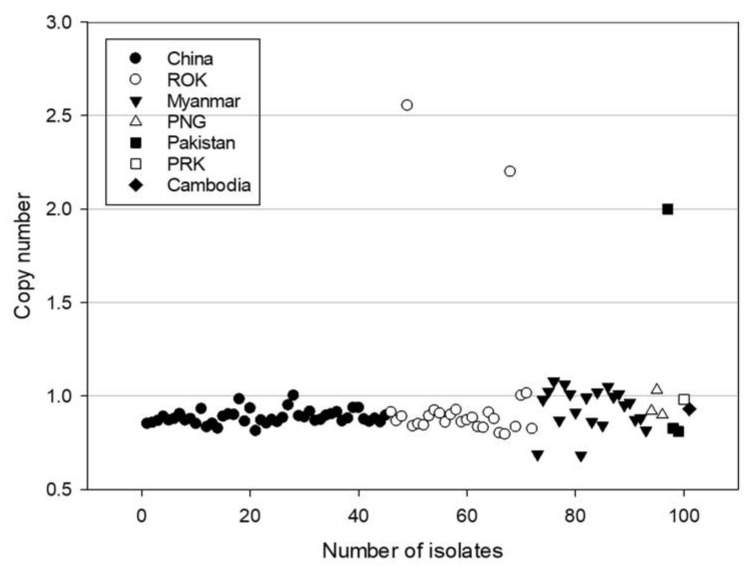
A scatter-gram depicting *pvmrp1* copy number estimates for *P. vivax* isolates from seven countries. ROK, the Republic of Korea; PNG, Papua New Guinea; PRK, the Democratic People’s Republic of Korea.

In this study, the amplification of *pvmrp1* was determined by the relative CN, which was calculated by the standard curve method. Except for one isolate from China, the CN of *pvmrp1* was assessed successfully from the other 101 isolates. The estimates of *pvmrp1* CN for these isolates ranged from 0.68 to 2.55. Most of the isolates carried one copy of the gene, and only three isolates had double CNs, two from the ROK and one from Pakistan (Figure 2). 

### 3.3. Correlation between Polymorphisms and In Vitro Drug Susceptibilities

Of the 102 sequenced isolates mentioned above, partial samples from China and ROK were tested for antimalarial drugs susceptibility in the previous study [20,21]. Combined, a total of 39, 34, 39, and 13 isolates were cultured for more than 24 h and assayed for the susceptibility to CQ, QN, MQ and pyrimethamine (PYR), respectively (Table S1, Figure S1). For CQ, the geometric mean IC_50_ was 20.92 nM (95% CI: 13–33.67 nM), with 5.12% (2/39) isolates revealing a resistant phenotype with IC_50_ > 220 nM. Furthermore, the IC_50_ of MQ was 10.02 nM (95% CI: 5.97–16.83 nM), the QN was 41.94 nM (95% CI: 24.82–70.88 nM), and the PYR was 43.22 nM (range: 19.26–96.98 nM). Within the sample set of isolates, the observed 13 non-synonymous *pvmrp1* SNPs (K36Q, T234M, T259R, R281K, S354K, K448I, E787D, Q906E, G949D, K1219N, Y1393D, V1478I, and H1586Y), did not appear to be correlated with the IC_50_ values of the four antimalarial drugs (Table S2). Using an IC_50_ value ≤ 220 nM as the sensitivity standard, the chi-square analysis was performed to analyze the correlation between the CQ sensitive/insensitive isolates and the mutation sites, the results showed that only the prevalence of S354N between these two CQ sensitivity categories revealed a significant difference (*p* < 0.05; Table 3). With regard to the variation of *pvmrp1* CN, increased *pvmrp1* CN did not appear to significantly alter parasites’ susceptibilities to CQ, MQ and QN (Figure S2).

### 3.4. The Polymorphism of pvmrp1 from Different Regions

To estimate the degree of genetic differentiation of the *pvmrp1* in global isolates, the sequences of *pvmrp1* obtained from the studied regions were compared with homologous sequences from the PlasmoDB database. An additional 72 *pvmrp1* sequences from PlasmoDB were downloaded for further analysis, including Myanmar (*n* = 4), PNG (*n* = 4), PRK (*n* = 1), Columbia (*n* = 21), Peru (*n* = 16), Mexico (*n* = 13), Thailand (*n* = 7), Brazil (*n* = 3), India (*n* = 2) and Mauritania (*n* = 1). All sequences were aligned and cut to 4095 bp (1087–5181 bp) by MEGA7.0. An analysis of the polymorphism of *pvmrp1* within the 166 global isolates revealed low nucleotide diversity (π = 0.0011) and high haplotype diversity (*Hd* = 0.9290). We also found significant differences in the gene polymorphism of *pvmrp1* in different regions. Get rid of Africa, the polymorphism of *pvmrp1* gene was the highest in South Asia (π = 0.0015), and the lowest in Southeast Asia (π = 0.0006). These results suggested that *pvmrp1* in different regions was subjected to different natural selection pressure and showed different levels of gene polymorphism (Table 4). 

### 3.5. Natural Selection of Polymorphic Region of pvmrp1 from Different P. vivax Isolates

To determine whether natural selection promoted the generation of *pvmrp1* gene diversity in global isolates, we calculated the ratio of non-synonymous to synonymous mutations (dn/ds). The dn/ds for *pvmrp1* of all the 166 isolates was 0.5536, indicating that the *pvmrp1* gene was affected by purifying selection. The overall Tajima’s D test value for *pvmrp1* of all the isolates was negative (Tajima’s D = −1.1863, *p* > 0.1), and the Fu and Li’s D* test of all the isolates was −3.9871 (*p* < 0.02) (Table 4). It suggested that a neutral model of polymorphism occurrence with values for *pvmrp1* was due either to a recent population expansion or genetic hitchhiking. In addition, we found that the significant Fu and Li’s D* test value < 0 (0.01 < *p* < 0.05) were found in isolates from South America and Southeast Asia. 

### 3.6. Genetic Differentiation, Haplotype Network and LD Analysis of Polymorphic Region of pvmrp1

The level of genetic differentiation of *pvmrp1* was estimated by F_ST_ values. As there is only one isolate from Africa, it was not considered. A low level of genetic differentiation was found in the isolates from South America and South Asia (the value of F_ST_ was 0.0809), while others were in a moderate and high level of genetic differentiation (0.1661–0.5498). This was especially true of the southeast Asian isolates, which showed a great genetic differentiation based on the values of F_ST_ (Table 5).

**Figure 3:**
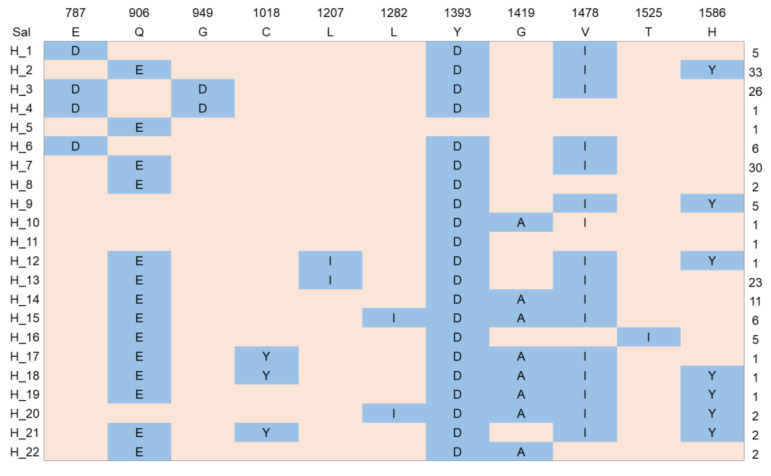
Amino acid alignment of the 22 *pvmrp1* haplotypes in 166 isolates. Polymorphic amino acids are listed for each haplotype. Amino acid residues are identical to the reference sequence *Sal*-I marked in pink. The dimorphic amino acid changes are marked in blue. The total number of sequences for each haplotype is listed in the right panel.

**Figure 4:**
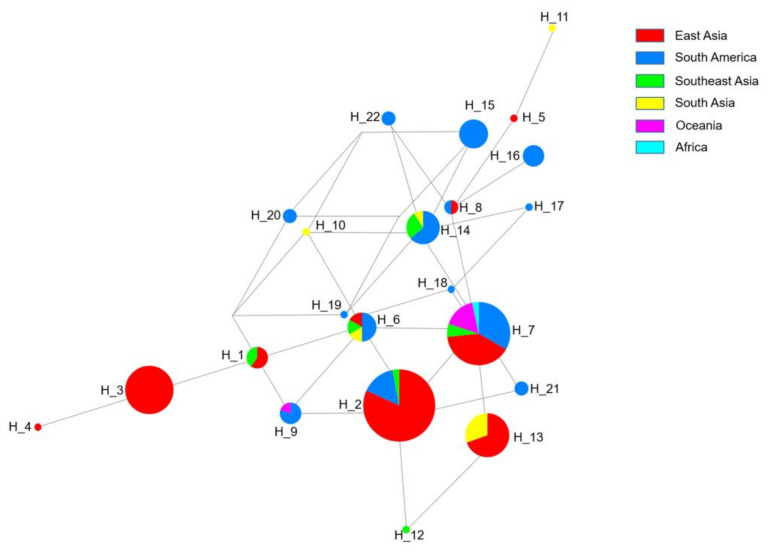
The haplotype network was created using 166 *pvmrp1* nucleotide sequences by the median-joining method in the NETWORK v5.0 program. The size of the circles indicates the haplotype frequency, and the color shows the geographical origin of the sequences. The lines between haplotypes display mutational steps.

To further verify the differential selection between groups, the 11 non-synonymous SNPs (E787D, Q906E, G949D, C1018Y, L1207I, L1287I, Y1393D, G1419A, V1478I, T1525I, and H1586Y), which occurred twice without deletion, were selected to construct a haplotype network among all 166 samples. The haplotype analysis of *pvmrp1* in this study revealed 22 distinct haplotypes (Figure 3). Eight of the haplotypes were singleton haplotypes, of which H_4 and H_5 were found in East Asian isolates exclusively, H_10 and H_11 existed only in South Asian isolates, H_12 was found in Southeast Asia, and H_17, H_18, and H_19 were found in South America specifically. The mutant types H_2: E**E**GCLL**D**G**I**T**Y** (18.7%) and H_7: E**E**GCLL**D**G**I**TH (18.1%) were the most common. H_2, H_3 (**D**Q**D**CLL**D**G**I**TH), and H_13 (E**E**GCLL**DA**ITH) were the dominant haplotype in East Asia (81.8%, 100.0%, and 69.6%, respectively), H_14 (**D**QGCLL**D**GVTH) is mainly distributed in South America (63.6%), and H_7 is distributed worldwide, which mainly includes East Asia (40%), South America (33.3%) and Oceania (16.6%) (Figure 4, Table S3). In particular, 14 haplotypes found in the isolates from South America, which indicated the highest haplotype diversity, and nine haplotypes in East Asia, which was consistent with the results of haplotype diversity in Table 4. 

**Figure 5:**
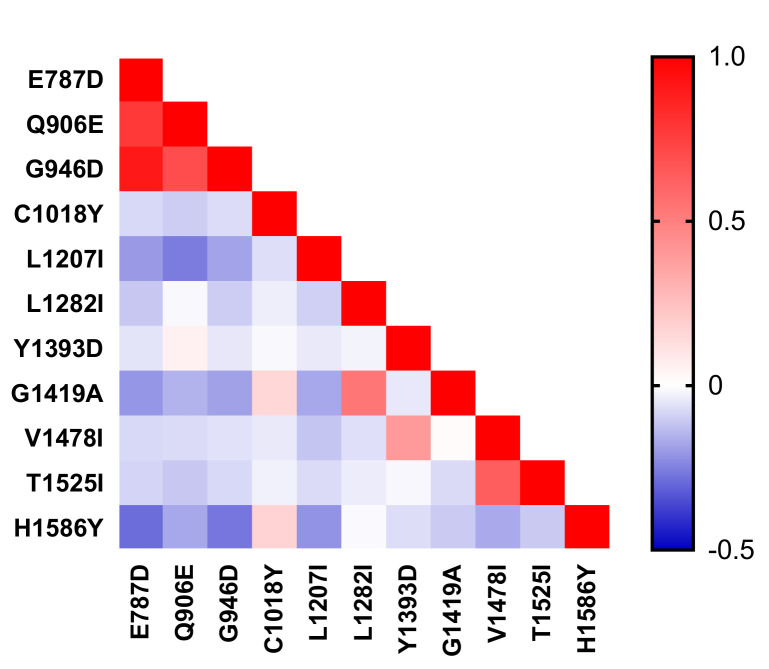
Linkage disequilibrium (LD) plot showing non-random association between nucleotide variants among 166 *P. vivax* isolates at 11 non-synonymous SNPs. R for each pair of genetic polymorphisms of *pvmrp1*.

In addition, by pairwise LD of the *pvmrp1* gene at 11 non-synonymous SNPs using the DNASP v5.10.01 program, we observed that there was a strong LD between E787D and Q906E, G949D, and H1586Y (*p* < 0.0001) (Figure 5). Meanwhile, the strong LD was present in *pvmrp1* gene between G949D and H1586Y, Q906E (*p* < 0.0001), and existed in the following pairs also: Q906E/L1207I, V1478I/T1525I (*p* < 0.0001). We found that most of the genes with LD occurred around the ABC transporter domain, such as E787D and Q906E, G949D.

**Figure 6:**
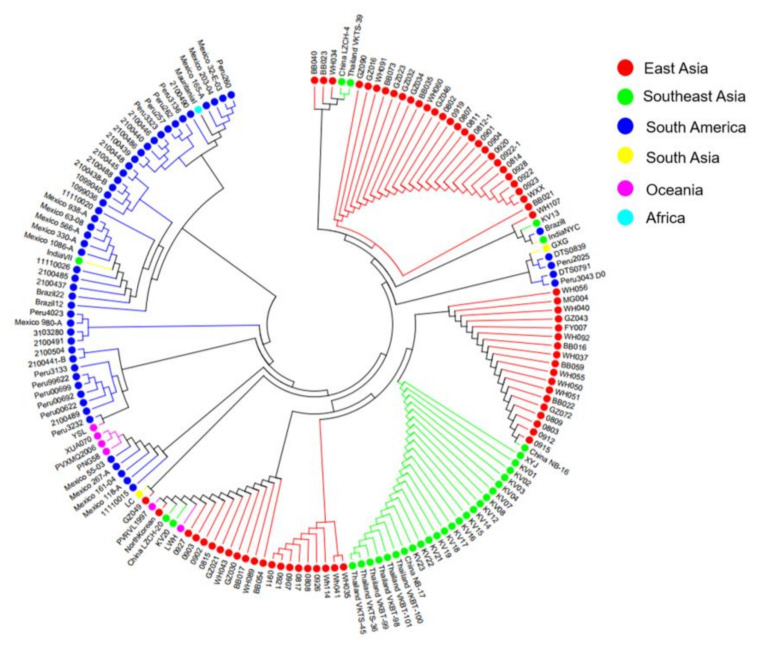
Phylogenetic relationship of *pvmrp1* full-length genes in 166 isolates based on the neighbor-joining method. The evolutionary history was inferred using the neighbor-joining method. The optimal tree with the sum of branch length = 51.8479 is shown. The evolutionary distances were computed using the number of differences method and are in the units of the number of base differences per sequence. The analysis involved 166 nucleotide sequences. All positions containing gaps and missing data were eliminated. There were a total of 3373 positions in the final dataset. The color shows the geographical origin of the sequences.

Furthermore, phylogenetic analysis of the 166 isolates indicated that the genetic evolution of *pvmrp1* varies in different regions, which have obvious population genetic structures related to geographical isolates. The genetic differentiation of *P. vivax* isolates indicated that the geographically distant was high; for example, the genetic differences between *pvmrp1* gene from East Asia and South America were the highest (Figure 6).

## 4. Discussion and Conclusions

*Plasmodium vivax* is the most geographically widespread cause of human malaria. Due to the lack of an in vitro culture and transgenic system, molecular markers represent a more practical tool to monitor the introduction and spread of drug resistance in *P. vivax* [31]. In our study, as a member of ABC transporters, PvMRP1, localizes at the parasite plasma membrane, and the predicted primary protein structure includes 11 TM and 2 NBDs, which function primarily as drug transports [32]. Consistent with the potential involvement of PvMRP1, it has been observed as a transporter with a broad range of substrates, including important endogenous substances such as glutathione and a lot of drugs with diverse structures. CQ was identified as a substrate for this ABC transporter, and the mutations of *pfmrp1* were found to be associated with reduced susceptibility to CQ and also to QN [13]. In addition, *pvmrp1* transcription level is decreased in the trophozoite stage, which is consistent with the phenotype that *P. vivax* trophozoites insensitive to CQ [33]. All of these suggest that *pvmrp1* may be a potential molecular marker of drug resistance.

In this study, we amplified and sequenced the *pvmrp1* gene of 102 whole blood samples originating from Asia, and 29 genetic mutations were found out of the 94 successfully sequenced isolates. Among them, most mutations have not been hitherto reported, and most of these mutations are found only in East Asia, which is likely due to the low number of east Asian isolates used in previous studies. The mutations T259R (97.87%), Y1393D (97.87%) and V1478I (95.74%) were approaching fixation in the sequenced samples, and the mutations V879 (89.5%) and L1207I (89.5%) were highly prevalent and present in Southeast Asia exclusively. A small number of imported isolates, such as South Asia (*n* = 2) and Oceania (*n* = 2) were not considered. We found significant differences in the types of mutations prevalent in East and Southeast Asia except for the mutations approaching fixation. A sequence similarity analysis of *pvmrp1* and *pfmrp1* indicated that the Y1393D and G1419A mutations of *pvmrp1* overlap with *pfmrp1* locations residing between the ABC transmembrane and the second ABC transporter domains which were associated with drug resistance [10]. Furthermore, a recent study has provided evidence that G1419A and V1478I had a significant association with the IC_50_ to CQ and artesunate, and G1419A was also associated with the decreased susceptibilities to PPQ, MQ, and QN [34]. However, in combination with our previous in vitro drug susceptibility studies [20,21], we could not find a correlation between the polymorphisms of *pvmrp1* and antimalarial drug susceptibilities (*p* > 0.05). The isolates used in our previous drug susceptibility studies, Y1393D and V1478I, were fixed, and no G1419A was observed in the *pvmrp1* gene, which may limit the correlation analysis. Moreover, we found that the prevalence of S354N between the two CQ sensitivity categories revealed a significant difference by chi-square analysis, although more clinical samples are needed to confirm this conclusion. Numerous studies have shown that gene CN polymorphism is related to genetic and phenotypic variation, which is as important as SNPs [35,36]. Three CNVs were also determined from two ROK isolates and one Pakistan isolate in this study, which indicates the variation in *pvmrp1* gene amplification in Asian isolates. However, we did not find a statistically significant correlation between CNVs and drug sensitivity.

The 94 sequenced samples combined with the 72 known sequences from the PlasmoDB database formed *pvmrp1* sequences worldwide. The result displayed a low genetic diversity at the *pvmrp1* with an average π of 0.0011. Haplotype analysis of *pvmrp1* showed that haplotype diversity varies in different regions, and haplotype polymorphism was higher in South America and Oceania than in the other areas. These results suggested that *pvmrp1* of *P. vivax* from different areas was subjected to various natural selection pressures and showed different levels of gene polymorphism and haplotype polymorphism. In this study, the rates of non-synonymous mutation to synonymous mutation of *pvmrp1* were < 1, and the neutral evolutionary test statistic Tajima’s D test was negative (Tajima’s D = −1.1863, *p* > 0.1), indicating that the *pvmrp1* gene was affected by purifying selection [27]. Fu and Li’s D* test further confirmed that the *pvmrp1* gene was under purifying selection (Fu and Li’s D* = −3.9871, *p* < 0.02), which suggested that the mutations and accumulate at silent sites, there were likely to be lots of segregating sites, but not much heterozygosity. This could explain that nucleotide diversity (π) was small and the average number of nucleotide differences (K) was high in Table 4.

Furthermore, genetic differentiation, the haplotype network, and phylogenetic analysis of the 166 isolates indicated that the genetic evolution of *pvmrp1* varies in different regions, which has obvious population genetic structures related to geographical isolates. The genetic differentiation between *P. vivax* isolates that were geographically distant was high, for example, the genetic differences between the *pvmrp1* gene from Southeast Asia and South America were the highest. Perhaps parasite movement could be controlled by different factors, including geographical barriers, distance, poor road infrastructure, cultural and language barriers, and the effectiveness of malaria control interventions. 

Overall, PvMRP1 localizes on the *P. vivax* plasma membrane with 11 TM, the gene nucleotide polymorphism, and CNVs were analyzed with the field *P. vivax* isolates in our study. We found some unreported point mutations of *pvmrp1* in the collected samples, and S354N substitution may lead to CQR in *P. vivax*. In combination with the worldwide isolates, the analysis showed that the *pvmrp1* gene had a high haplotype diversity, low nucleotide diversity and was under purifying selection. This study showed the polymorphisms of the *pvmrp1* gene from worldwide isolates, and pointed to the potential contribution of the *pvmrp1* gene in the CQR of *P. vivax*. However, the lack of correlation between the *pvmrp1* polymorphisms and the IC_50_ values of the four antimalarial drugs (CQ, MQ, QN and PYR), highlights the need for more informative tools to function the role of *pvmrp1* gene.

## Figures and Tables

**Table 1:** Sequences and features of primers used in nest/seminest PCR and Real-Time PCR.

Purpose	Primer Name	Role in PCR	Sequence (5′-3′)	Position	PCR Product (bp)	Annealing Temperature
Sequencing						
Fragment 1	Frag1-F1	First, second and Seq PCR	AAA AAA TAA GCC AAA TTA ACC TTA CAC	−48–−74	First round 2500 bp	56 °C
	Frag1-R1	First PCR	GGC AAA ATG CAC TTA TTT TGT CT	2430–2452		
	Frag1-R2	Second PCR	TTT ATG TTC TTC AAA ATG TAC TTC TCA	1926–1952	second round 2000 bp	56 °C
	Frag1-SF1	Seq PCR	GGA TTT TTT TTA ACA CTG TTA CAG CTT	726–752		
	Frag1-SR2	Seq PCR	GAT TTA ACG AAT CAT TCT GTG TAT AGA AA	652–680		
	Frag1-SR3	Seq PCR	CAT AGT TGA AGT GTT GTT ATT TTT TTT GT	1302–1330		
Fragment 2	Frag2-F1	First PCR	TGA TGT AGA GAA AGT GTG TTT CCA G	1752–1776	First round 2401 bp	61 °C
	Frag2-R1	First PCR	CTC CTC TAG CCT CTG CAC ACA	4132–4152		
	Frag2-F2	Second and Seq PCR	TTA GAA AAT GCT TTT TTT GGC AC	1882–1904	second round 1971 bp	56 °C
	Frag2-R2	Second PCR	TAT ACC TAA ATG GTA CCA ATT CTT TTC	3826–3852		
	Frag2-SF1	Seq PCR	AAT AAG AGC TTC AAA GAC TAT TGC AGT	2602–2628		
	Frag2-SF2	Seq PCR	TAT ATC TAT CTT TAC AGA TGA AAT AAA ATT	3252–3281		
Fragment 3	Frag3-F1	First PCR	CAG TGA AGG TGC ACA CAG ATG	3452–3472	First round 1792 bp	59 °C
	Frag3-R1	First, second and Seq PCR	TAC CAC ACG TCG AAC GTG G	+44–+62		
	Frag3-F2	Second and Seq PCR	TTA TGC AAC ATA TAT AGC AAC ACC AT	3772–3797	second round 1472 bp	56 °C
	Frag3-SF1	Seq PCR	TTA TTT TGT CCA TCT TAG GGC TC	4502–4524		
**CN detection**						
Reference gene	Pvtubulin–FP	Real-Time PCR	CAA GAA CTC CTC CTA CTT CGT CG	1644–1666	122 bp	58 °C *
	Pvtubulin–RP	Real-Time PCR	GTT GCG TGG AAA GCC ATC TC	1746–1765		
	Pvtubulin–P	Probe	HEX-TGCCCAACAGGGAGGAAGCGATT-BHQ1	1699–1721		
Target gene	PvMRP-FP	Real-Time PCR	AAA GTG TGT TTC CAG ACA AGA GTT	1762–1785	111 bp	58 °C
	PvMRP-RP	Real-Time PCR	CAA ATT GCT TCG CTC CTC TG	1853–1872		
	PvMRP-P	Probe	FAM-TGCTCCAATGGCGGCAGTAGTAGTAG-BHQ1	1789–1814		

* Reference [21].

**Table 2:** The prevalence of *pvmrp1* gene substitution in 94 parasite isolates.

NO.	AA Change	Effect	Number (%)	Frequency (%)			
				East Asia (*n* = 71)	Southeast Asia (*n* = 19)	South Asia (*n* = 2)	Oceania (*n* = 2)
1	N67S	non-synonymous mutation	1 (1.06)	1.4			
2	K36Q	non-synonymous mutation	1 (1.06)	1.4			
3	T234M	non-synonymous mutation	18 (19.15)	1.4	**89.5**		
4	T259R	non-synonymous mutation	92 (97.87)	**100**	**100**	**100**	
5	F271Y	non-synonymous mutation	1 (1.06)			**50**	
6	R281K	non-synonymous mutation	11 (11.70)	15.5			
7	T282M	non-synonymous mutation	1 (1.06)			**50**	
8	S354N	non-synonymous mutation	9 (9.57)	12.7			
9	F406L	non-synonymous mutation	1 (1.06)	1.4			
10	K448I	non-synonymous mutation	1 (1.06)	1.4			
11	E533	gene deletion	3 (3.19)	4.2			
12	F560I	non-synonymous mutation	1 (1.06)			**50**	
13	N568	synonymous mutation	2 (2.13)			**50**	**50**
14	E787D	non-synonymous mutation	30 (31.9)	42.3			
15	C817	synonymous mutation	1 (1.06)				
16	A853	synonymous mutation	20 (21.28)	28.2			
17	V879	synonymous mutation	17 (18.09)		**89.5**		
18	Q906E	non-synonymous mutation	59 (62.77)	**56.3**	**94.7**		**50**
19	G949D	non-synonymous mutation	27 (28.72)	38.0			
20	E967G	non-synonymous mutation	1 (1.06)		5.3		
21	L1207I	non-synonymous mutation	17 (18.09)		**89.5**		
22	K1219N	non-synonymous mutation	2 (2.13)	2.8			
23	I1232	synonymous mutation	38 (40.43)	28.2	**89.5**	**50**	
24	Y1393D	non-synonymous mutation	92 (97.87)	**98.6**	**100**	**50**	**100**
25	V1360	non-synonymous mutation	9 (9.57)	12.7			
26	G1419A	non-synonymous mutation	1 (1.06)			**50**	
27	V1478I	non-synonymous mutation	90 (95.74)	**95.8**	**100**	**50**	**100**
28	H1586Y	non-synonymous mutation	29 (30.85)	38.0	5.3		**50**

Allele frequency bolded if >50%.

**Table 3:** Comparison of *pvmrp1* substitutions between CQ sensitive and CQ resistant isolates.

Mutations		CQ Sensitive (*n*) ^a^	CQ Resistant (*n*) ^a^	*p*-Value *
E787D	D	16	1	0.2491
	E	22	0	
G949D	D	15	1	0.2245
	G	23	0	
Q906E	E	20	1	0.3483
	Q	18	0	
H1586Y	Y	14	1	0.2
	H	24	0	
S354N	N	6	1	0.0303 *
	S	32	0	

^a^ Using 220 nM as the cutoff IC_50_ for CQ resistance. >220 nM was considered as CQ resistant isolates, and ≤220 nM were considered as CQ sensitive isolates. * indicates a significant correlation.

**Table 4:** Estimates of the number of isolates and SNPs, nucleotide diversity, haplotype diversity and neutrality indices of *pvmrp1*.

Region	*n*	S	η	H	*Hd*	π	k	dn/ds	Tajima’s D	*p*-Value	Fu and Li’s D*	*p*-Value
South America	53	24	25	28	0.9544	0.0009	3.4933	0.7748	−1.1743	*p* > 0.1	−2.0414	0.05 < *p* < 0.1
East Asia	72	11	11	15	0.8581	0.0008	3.2021	0.5630	1.1341	*p* > 0.1	0.8084	*p* > 0.1
Southeast Asia	30	15	15	8	0.4180	0.0006	2.0172	0.6154	−1.5484	*p* > 0.1	−2.0390	0.05 < *p*< 0.1
South Asia	4	10	10	4	1.0000	0.0012	5.0000	0.4009	0.2982	*p* > 0.1	0.2982	*p* > 0.1
Oceania	6	10	10	6	1.0000	0.0009	3.3333	0.6762	−0.9837	*p* > 0.1	−1.1374	*p* > 0.1
Africa	1	6	6	1	1.0000	0.0015	6.0000	-	-	-	-	-
Total	166	34	35	46	0.9290	0.0011	3.6571	0.5536	−1.1863	*p* > 0.1	−3.9871	*p* < 0.02

*n*: number of sequences; S: number of polymorphic sites; η: total number of mutations; H: number of Haploytypes; *Hd*: Haplotype diversity; π: nucleotide diversity; K: average number of nucleotide differences; dn: the rates of non-synonymous substitutions; ds: the rates of synonymous substitutions.

**Table 5:** Estimation of genetic differentiation (F_ST_ *) of the *pvmrp1* among geographical populations.

Population	East Asia	South America	Southeast Asia	South Asia	Oceania
East Asia (*n* = 72)	0.0000				
South America (*n* = 53)	0.3788	0.0000			
Southeast Asia (*n* = 30)	0.4832	0.5498	0.0000		
South Asia (*n* = 4)	0.1957	0.0809	0.3682	0.0000	
Oceania (*n* = 6)	0.2350	0.1589	0.5351	0.1661	0.0000

* the value of F_ST_ (0.05–0.15) is poor differentiation, F_ST_ (0.15–0.25) is moderate differentiation, and F_ST_ > 0.25 is great differentiation.

## Data Availability

Data is contained within the article.

## Supplementary Materials

The following supporting information can be downloaded at: https://www.mdpi.com/article/10.3390/microorganisms10081482/s1, Table S1: IC_50_ values to four antimalarial drugs of *P. vivax* isolates; Figure S1: Dot plots of in vitro susceptibilities of *P. vivax* isolates to four antimalarial drugs; Table S2: Association of SNPs in *pvmrp1* with in vitro susceptibilities to CQ, MQ, QN and PYR; Figure S2: Comparison of IC_50_ values for three antimalarials between parasites with single copy and multicopies of *pvmrp1* gene; Table S3: Distribution of the number of 22 *pvmrp1* haplotypes in worldwide isolates; Spreadsheet S1: Sample information.

## Sample Availability

Samples of the compounds are available from the authors.
