# Supplementary material for: Prevalence of pvmrp1 Polymorphisms and Its Contribution to Antimalarial Response
Source: Microorganisms. 2022 Jul 22;10(8):1482. doi: 10.3390/microorganisms10081482 (PMC9394237; doi:10.3390/microorganisms10081482)
Supplement: Supplementary file 1 [file microorganisms-10-01482-s001.zip › microorganisms-1816917-supplementary/supplementary materials/Supplementary Materials.pdf]

Table S1. IC_{50} values to four antimalarial drugs of *P. vivax* isolates.

Drugs	Number	Mean	95% CI
CQ	39	20.92	13 - 33.67
MQ	34	10.02	5.97 - 16.83
QN	39	41.94	24.82 - 70.88
PYR	13	43.22	19.26 - 96.98

CQ, chloroquine; MQ, mefloquine; QN, Quinine; PYR, pyrimethamine.

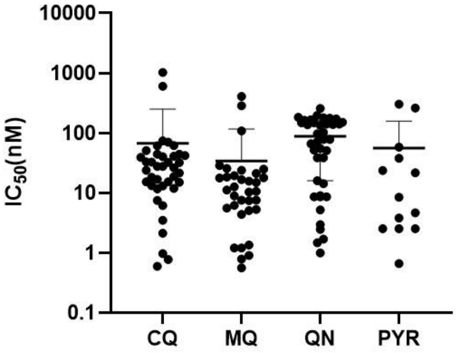

Figure S1. Dot plots of *in vitro* susceptibilities of *P. vivax* isolates to four antimalarial drugs.

Table S2. Association of SNPs in *pvmrp1* with *in vitro* susceptibilities to CQ, MQ, QN and PYR.

Mutations		*n*	CQ IC_{50} (nM)		*p value*
			Geometric mean	95% CI	
K36Q	Q	1	0.9692	-	-
	K	38	22.68	14.33-35.90	
T259R	R	39	20.92	13.00-33.67	-
	T	0	-	-	
R281K	K	4	8.924	1.243-64.05	0.1345
	R	35	23.06	13.89-38.29	
S354N	N	7	29.89	4.355-205.1	0.5977
	S	32	19.35	11.98-31.25	
K448I	I	1	0.7772	-	-
	K	38	22.81	14.48-35.95	
E787D	D	17	28.09	15.57-50.67	0.428
	E	22	16.66	7.959-34.86	
Q906E	E	21	16.59	7.627-36.07	0.4988
	Q	18	27.42	11.83-35.60	
G949D	D	16	32	18.32-55.91	0.2197
	G	23	15.56	7.592-31.90	
K1219N	N	1	3.491	-	-
	K	38	21.93	13.58-35.41	
Y1393D	D	39	20.92	13.00-33.67	-
	Y	0	-	-	
V1478I	I	39	20.92	13.00-33.67	-
	V	0	-	-	
H1586Y	Y	15	24.03	9.820-58.80	0.5067
	H	24	19.18	10.63-34.61	

Mutations		n	QN IC_{50} (nM)		*p value*
			Geometric mean	95% CI	
K36Q	Q	1	2.468	-	-
	K	38	45.19	26.96-75.73	
T234M	SM	1	40.35	23.68-68.76	-
	T	38	183.2	-	
T259R	R	39	41.94	24.82-70.88	-
	T	0	-	-	
R281K	K	5	19.01	1.423-253.8	0.4708
	R	34	47.12	27.51-80.72	
S354N	N	3	149.2	117.1-190.0	0.1543
	S	36	37.74	21.66-65.76	

E787D	D	18	30.93	13.58-70.45	0.1759
	E	21	54.48	26.55-111.8	
Q906E	E	21	54.46	26.54-111.7	0.1805
	Q	18	30.93	13.58-70.45	
G949D	D	16	48.64	15.07-76.31	0.2536
	G	23	45.54	23.32-101.4	
K1219N	N	6	38.27	6.501-225.3	0.5267
	K	33	42.65	23.91-76.07	
Y1393D	D	38	40.35	23.68-68.76	-
	Y	1	183.2	-	
V1478I	I	38	40.35	23.68-68.76	-
	V	1	183.2	-	
H1586Y	Y	15	51.24	21.11-124.4	0.5536
	H	24	37.01	18.44-74.30	

Mutations		*n*	PYR IC50（nM)		*p value*
			Geometric mean	95% CI	
T259R	R	13	43.22	19.26-96.98	-
	T	0	-	-	
R281K	K	2	103.2	0.00007852-135630097	0.4103
	R	11	36.9	15.28-89.08	
E787D	D	8	30.5	9.097-102.3	0.2844
	E	5	75.49	20.90-272.7	
Q906E	E	5	75.49	20.90-272.7	0.2844
	Q	8	30.5	9.097-102.3	
G949D	D	6	32.6	12.01-88.50	0.366
	G	7	55.04	12.13-249.8	
K1219N	N	2	24.98	1.104e-011-56508089506048	0.9231
	K	11	47.75	23.81-95.77	
Y1393D	D	13	43.22	19.26-96.98	-
	Y	0	-	-	
V1478I	I	13	43.22	19.26-96.98	-
	V	0	-	-	
H1586Y	Y	3	117.5	7.071-1953	0.1608
	H	10	32.02	12.71-80.63	

Mutations		n	MQ IC_{50} (nM)		*p value*
			Geometric mean	95% CI	
K36Q	Q	1	1.205	-	-
	K	33	10.68	6.366 - 17.93	
T259R	R	34	10.02	5.966 - 16.83	-
	T	0	-	-	
R281K	K	3	9.389	0.1127 - 782.0	0.5272
	R	31	10.08	5.840 - 17.41	
S354N	N	7	15	10.03 - 22.44	0.2038
	S	27	9.024	4.709 - 17.29	
K448I	I	1	18	-	-
	K	33	9.843	5.772 - 16.78	
E787D	D	15	12.36	4.605 - 33.20	0.8173
	E	19	8.478	4.691 - 15.32	
Q906E	E	19	8.487	4.696 - 15.34	0.8036
	Q	15	12.36	4.605 - 33.20	
G949D	D	14	9.877	3.886 - 25.10	0.4414
	G	20	10.11	5.175 - 19.76	
K1219N	N	1	287	-	-
	K	33	9.051	5.541 - 14.78	
Y1393D	D	34	10.02	5.966 - 16.83	-
	Y	1	1.205	-	

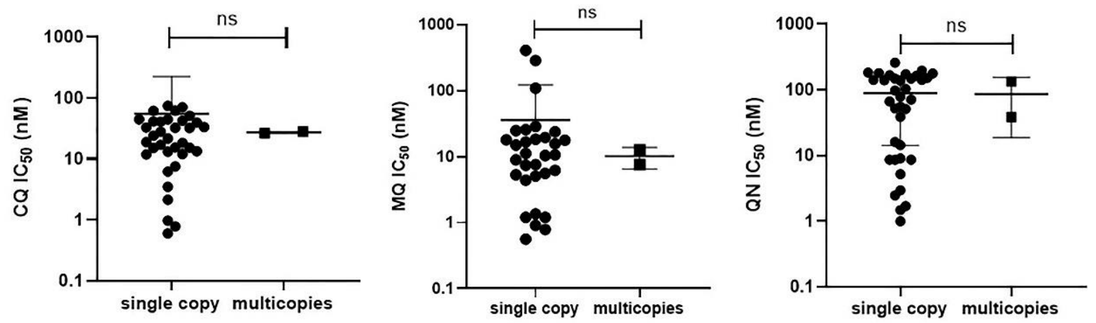

Figure S2. Comparison of IC_{50} values for three antimalarials between parasites with single copy and multicopies of *pvmrp1* gene. ns: no significance.

Table S3. Distribution of the number of 22 *pvmrp1* haplotypes in worldwide isolates.

ID	Count	South America					East Asia				Southeast Asia				South Asia			Oceania	Africa
		Brazil	Columbia	Mexico	Peru	Total	China	ROK	PRK	Total	Myanmar	Cambodia	Thailand	Total	Pakistan	India	Total	PNG	Mauritania
H_1	5						3			3	1		1	2					
H_2	33		1	4		5	17	10		27	1			1					
H_3	26						12	13	1	26									
H_4	1						1			1									
H_5	1						1			1									
H_6	6	1	2			3	1			1	1			1		1	1		
H_7	30		2	7	1	10	7	4	1	12	2			2				5	1
H_8	2				1	1	1			1									
H_9	5				4	4												1	
H_10	1														1		1		
H_11	1														1		1		
H_12	1											1		1					
H_13	23							16		16					1	6	7		
H_14	11	2		5		7						3		3		1	1		
H_15	6		6			6													
H_16	5		4		1	5													
H_17	1		1			1													
H_18	1		1			1													
H_19	1		1			1													
H_20	2				2	2													
H_21	2			1	1	2													
H_22	2				2	2													
